# Exploring the therapeutic potential of localized alpha irradiation for cancer: from DNA damage to immune activation

**DOI:** 10.1093/bjro/tzaf030

**Published:** 2025-11-24

**Authors:** Saskia Hazout, Daniel Zwahlen, Christoph Oehler, Ambroise Champion, David Benzaquen, Daniel Taussky

**Affiliations:** Department of Radiation Oncology, Centre Hospitalier de l’Université de Montréal—CHUM, Montreal, QC H2X 0C1, Canada; Department of Radiation Oncology, Kantonsspital Winterthur, Winterthur, ZH 8401, Switzerland; Department of Radiation Oncology, Kantonsspital Winterthur, Winterthur, ZH 8401, Switzerland; Radiation Oncology, Hôpital de La Tour, Meyrin, Meyrin, GE 1217, Switzerland; Radiation Oncology, Hôpital de La Tour, Meyrin, Meyrin, GE 1217, Switzerland; Department of Radiation Oncology, Centre Hospitalier de l’Université de Montréal—CHUM, Montreal, QC H2X 0C1, Canada; Radiation Oncology, Hôpital de La Tour, Meyrin, Meyrin, GE 1217, Switzerland

**Keywords:** alpha irradiation, LET, DSB, DaRT

## Abstract

Alpha radiation has emerged as a promising modality in cancer treatment due to its unique physical and biological properties. Among these, diffusing alpha-emitters radiation therapy (DaRT) delivers alpha radiation directly into solid tumours using inserted seeds. This review synthesizes both the biological mechanisms and therapeutic implications of alpha irradiation, with a focus on DaRT. We explore how alpha particles induce complex DNA damage, modulate the tumour microenvironment, and interact with immune therapies. Emphasis is placed on preclinical and early clinical findings that suggest DaRT’s potential to improve outcomes, especially in difficult-to-treat malignancies. The high linear energy transfer (LET) radiation induces complex DNA damage in tumour cells, leading to increased cell death compared to conventional radiotherapy. Alpha particles have a short range in tissue, allowing for highly localized treatment with minimal damage to surrounding healthy tissue. Recent studies have demonstrated that alpha radiation can stimulate antitumor immune responses, potentially enhancing treatment efficacy. Clinical trials utilizing alpha-emitting radioisotopes have shown encouraging results in various cancer types, particularly for metastatic disease.

## Introduction

Diffusing alpha-emitter radiation therapy (DaRT) is an innovative approach to cancer treatment that involves the direct delivery of alpha radiation to solid tumours using manually inserted seeds. This technique has garnered attention due to its potential to enhance the efficacy of immunotherapy. The unique properties of alpha radiation, including its high linear energy transfer (LET) and greater biological effectiveness compared to low-LET radiation such as X-rays and gamma rays, make it a promising candidate for cancer treatment.

Historically, the increased toxicity of radium, an alpha- and beta-emitter, compared to X-rays was observed by clinicians. This observation laid the foundation for understanding the superior efficacy of alpha irradiation in cancer treatment.^1^ The biological damage caused by high-LET radiation significantly surpasses that of low-LET radiation, making alpha particles particularly effective in destroying cancer cells. In recent years, there has been a notable increase in interest in alpha irradiation, despite challenges such as the short half-lives of radionuclides, limited availability of isotopes, and the complex requirements for delivery to solid tumours. This renewed interest is driven by both scientific advancements and clinical progress. There has been a surge in publications and funding for alpha therapy research. Furthermore, the FDA approved Xofigo (^223^Ra) for metastatic prostate cancer in 2013. This validated alpha therapy as a viable clinical modality and spurred further investment into next-generation approaches like DaRT. Furthermore, given that alpha particles effectively induce immunogenic cell death (ICD), the integration of alpha-emitter therapies with immune checkpoint inhibitors presents significant potential for enhanced antitumor efficacy through synergistic interactions.^2^

The theoretical advantages of alpha irradiation appear promising in this regard. Clinical experience with alpha radiation in nuclear medicine includes targeted α-therapy (TAT) using monoclonal antibodies (mAbs) and the calcium analogue 223Ra (Xofigo) have been successful.

Compared to the localized approach of DaRT, these systemic therapies have limitations in terms of specificity and accumulation in larger tumours.

Recent studies have focused on elucidating the basic science behind the effects of alpha irradiation.^3^^,^^4^ This review aims to explore the scientific basis for the theoretical advantages of alpha irradiation and especially of the DaRT seeds as a local, nonsystemic treatment. We examine preclinical and clinical evidence supporting the use of manually-placed seeds containing alpha radiation for local treatment. Our analysis draws primarily from studies on the systemic application of alpha-emitters to provide a comprehensive understanding of this emerging therapeutic approach.

## Methods

To write this review, the PubMed and Google Scholar databases were consulted. We searched for articles regarding alpha irradiation as a localized and not systemic treatment. To achieve this objective, we conducted a review of scholarly articles to identify original research that specifically examined the effects of alpha irradiation. Particular emphasis was placed on studies that compared the effects of alpha irradiation with those of conventional low-LET treatments, such as X-rays, γ-rays, and β-emitters. Although we admit that this is a subjective definition, we focused on clinically relevant data. We prioritized studies that directly compared alpha-particle irradiation with low-LET modalities (X-rays, γ-rays, and β-emitters) to elucidate the unique radiobiological effects of alpha irradiation in contrast to other irradiation modalities, as well as articles with clinical significance. This article is not a systematic review but rather a narrative synthesis of recent literature on alpha irradiation, with an emphasis on DaRT. Our aim was to focus on clinically relevant studies that compared the effects of alpha irradiation with other forms of radiation. These selections allowed us to highlight the distinctive biological features of high-LET radiation without drawing broad conclusions beyond the published evidence base.

## Results/discussion

**Figure 1.**
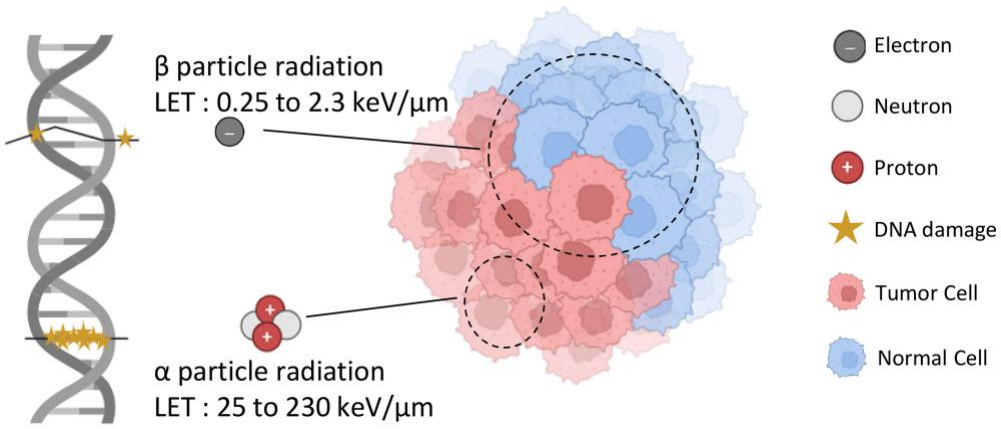
Illustration of high-LET radiation. This schematic contrasts high-LET and low-LET radiation modalities. Alpha particles (LET: 25-230 keV/μm) deposit dense ionizations over a short track, producing clustered, complex DNA damage within 5-10 cell diameters. Beta particles (LET: 0.25-2.3 keV/μm), electrons, protons, and neutrons create sparser ionizations along longer trajectories, yielding more isolated DNA lesions. The illustration highlights how densely ionizing alpha tracks inflict greater irreparable damage in tumour cells while sparing surrounding normal cells due to their limited range.

The studies reviewed reveal distinctive biological advantages of high-LET radiation (Figure 1), particularly alpha particles, Table 1 provides critical insight into cellular response to alpha-particle irradiation across cell types. Table 1 presents a compilation of pivotal *in vitro* studies examining alpha-particle irradiation across various cell types, including melanoma, lung fibroblasts, and human-hamster hybrid lines. Collectively, these investigations highlight several recurring themes: alpha particles necessitate only a limited number of traversals to achieve significant cell mortality; they induce complex, clustered DNA lesions that are resistant to repair; they generate reactive oxygen species (ROS) and mitochondrial stress even in the absence of direct nuclear impact; and they provoke non-targeted (bystander) effects in adjacent cells. These mechanistic insights provide the radiobiological rationale for DaRT’s localized, high-LET approach and are directly linked to its observed efficacy in preclinical tumour models. Several findings substantiate the mechanistic rationale of DaRT; for example, Pikul et al^5^ demonstrated that 5-7 decays per cell can achieve substantial cell kill in melanoma models. Studies by Lloyd et al^6^ and Narayanan et al^7^ identified direct nuclear effects and extranuclear targets, suggesting alpha-induced cytotoxicity involves ROS and mitochondrial stress pathways. DNA double-strand breaks (DSBs) induced by alpha particles are more complex and less repairable than gamma rays, with Franken et al^8^ and Carter et al^9^ confirming higher chromosomal rearrangement rates. Studies by Wu et al^10^ and Zhou et al^11^ indicate significant bystander and mutagenic effects beyond directly irradiated cells. These results support the high relative biological effectiveness (RBE) and non-local effects of alpha irradiation—qualities central to DaRT’s advantages. Unlike systemic alpha therapies limited by distribution and tumour penetration, DaRT offers targeted delivery of alpha particles to the tumour microenvironment, enabling local cytotoxicity and immune activation with minimal systemic toxicity. The DaRT treatment consists of delivering ^224^Ra seeded rods with a diameter of 0.7 mm manually with needles into the cancer. The advantages in terms of mechanism and radiobiological features of DaRT are its small diffusion radius (50-100 µm) therefore permitting to deliver higher doses to the cancer with better sparing of surrounding healthy tissue, a higher LET and therefore improved damage to the cancer and minimal oxygen dependency and therefore higher efficacy in hypoxic cancers. The ^224^Ra seeds release ^220^Rn gas, which diffuses into nearby tumour cells and decays to alpha-emitting progeny. This localized delivery of the seeds is a huge advantage compared to systemic alpha therapies because it permits to deliver the highly effective treatment to a very narrow part of tissue. These short-range alpha particles from DaRT induce dense ionization in tumour microregions as an additional benefit to the high-LET effect.

**Table 1.** *In vitro* studies investigating the effects of alpha-particle irradiation.

Authors	Ref	Cell lines	Results
Extranuclear/ROS and mitochondrial effects			
Wu et al	[61]	Mammalian cells	Irradiation of cellular cytoplasm with either a single or an exact number of α-particles results in gene mutation in the nucleus while inflicting minimal toxicity
Pikul et al	[5]	Melanoma cells	α-Particles achieved cell killing with relatively few atoms, 5-7 decays per cell resulted in a substantial cell kill (>85%)
Deshpande et al	[64]	Human diploid lung fibroblasts (HFL1)	There is the possible existence of an extranuclear compartment as a target for α particles
Lloyd et al	[6]	Mammalian cells	α-Particles needed 10 and 20 particles and not the expected 1 or 2 particles to cause a lethal effect in flattened cells
Wu et al	[10]	Human-hamster hybrid (A_L_) cells	Irradiation of the cytoplasm with α-particles is largely nonlethal; at least 8 or more particle traversals are needed. It is possible that nonnuclear traversals are indirectly mediated by ROS
Cell-cycle arrest and reproductive inactivation			
Lloyd et al	[63]	Mouse embryo fibroblasts	A total of 10-20 alpha particles needed to traverse the nucleus to produce 1 lethal event
Narayanan et al	[7]	Human lung fibroblasts	Alpha irradiation caused significant increases in intracellular O_2_·^−^ production, along with concomitant increases in H_2_O_2_ production. This means that alpha particles may mediate DNA damage through a reactive oxygen species (ROS)-related mechanism
Azzam et al	[65]	Human diploid fibroblasts	There was higher toxicity of alpha particles, as neither the repair of potentially lethal damage nor a reduction in the fraction of cells transiently or permanently arrested in G1 was observed compared to that in gamma-irradiated cells
DNA double-strand-break complexity			
Cheng et al	[66]	Human peripheral blood lymphocytes	The application of α-particles and X-rays showed that the DNA repair pathways were much more highly activated by α-particle irradiation than with the use of X-rays. There was an individual variability in the response to mixed beams
Pouget et al	[67]	Cells (sic)	The amount of cellular DNA damage was 2-fold lower after high-LET irradiation than after low-LET irradiation with gamma rays
Levy et al	[39]	Melanoma cells	Alpha irradiation was more lethal than photons
Rodriguez et al	[68]	Thyroid cells	Boron neutron capture therapy compared to photons shoed a higher number of DNA damage with photons but focus size larger with BNCT
Carter et al	[9]	HeLa and oropharyngeal squamous cell carcinoma	Alpha -particles increased complex DNA damage compared to low-LET protons or X-rays
Franken et al	[8]	Human squamous cell lung carcinoma derived line	Both alpha and gamma irradiation induce similar numbers of ionizing radiation induced foci (IRIFs) per absorbed dose. α-Particles, compared to gamma-rays, caused a considerably greater fraction of DNA double-strand breaks (DSBs) that result in chromosome rearrangements and cell reproductive death
Gadbois et al	[62]	Normal skin fibroblasts	α-Particles, compared to gamma radiation, were more potent per Gray in causing cell cycle effects and inactivating reproductive capacity Both α-particles and X-rays cause elevations in cellular p53 and p21, X-rays can induce the expression of an abundant amount of a lower-molecular weight form of p21 that is present only in very low amounts after alpha irradiation
Zhou et al	[11]	Human-hamster hybrid (A_L_) cells	Alpha irradiated cells can induce a bystander mutagenic response

The FDA has granted breakthrough device designation to Alpha DaRT for the treatment of patients with recurrent glioblastoma multiforme in 2021. In the following sections, we are focusing on the advantages of alpha irradiation and what is scientifically known and what still need to be further explored.

### Physics of alpha irradiation

Alpha particles are positively charged particles with a mass and charge equivalent to a helium nucleus (composed of 2 protons and 2 neutrons). Because the range of irradiation with alpha particles is small, healthy tissue can be better spared than with non-alpha radiation techniques. Alpha irradiation appears to be more deleterious, resulting in a greater RBE than irradiation with gamma rays, X-rays, or beta particles. Alpha particles, emitted by alpha-nuclides, are helium-4 nuclei (24 He2+) with a double positive charge, notable for their considerable mass and high-energy levels ranging from 4 to 9 MeV. This high LET allows a single alpha particle to cause irreparable DNA DSBs. Additionally, due to their substantial mass and charge, alpha particles interact more intensely with their surroundings, limiting their range in tissue to approximately 45-100 µm, or the diameter of 5-10 cells. Furthermore, the direct DSBs primarily caused by alpha particles makes it independent from oxygen free radicals from water radiolysis and its effect independent from the cell cycle phases.^12^

The decays of the ^224^Ra have an effect on the tissue around the seed through diffusion and convection. The advantage of the ^224^Ra is that there is a larger range than with most other alpha-emitting radionuclides. Charged particles, such as alpha particles also have a Bragg peak, meaning that the particle deposits more energy near the end of its path. This allows also a better predictability of the dosimetry.^13^ At the end of the decay there is a stable ^208^Pb atom. There are several Pb binding molecules that could affect the efficacy of the DaRT which should not be neglected.^13^

### Therapeutic applications of alpha radiation

Currently, there are several clinical applications for alpha radiation.^14^

1. ^223^Radium (^223^Ra, Xofigo) (Figure 3A) was the first alpha-emitting therapy proven effective. Presently, it is the only clinically and commercially available alpha-particle emitter,^15^ and it has been approved as a systemic intravenously injected treatment of metastatic prostate cancer. As a calcium analogue, ^223^Ra is used as a nonconjugated molecule that targets osteoblastic bone metastasis^16^ (Figure 3A).

^226^Ra, an alpha and gamma emitter discovered in 1898 by Marie Sklodowska-Curie and Pierre Curie, was formerly used for brachytherapy of most body areas, particularly for cervical cancer, but has been replaced since the 1950s by other sources, such as cobalt-60 and cesium-137. In treating metastatic castration-resistant prostate cancer (mCRPC) in the ALSYMPCA Phase III trial,^15^ Xofigo improved overall survival by 3.6 months versus placebo (14.9 versus 11.3 months), with a hazard ratio of 0.695 (*P* = .00185), and delayed first symptomatic skeletal event. Additionally, the PEACE III trial^17^ demonstrated combining Xofigo with enzalutamide improved PSA response and progression-free survival, reducing progression risk by 31%. The REASSURE study^18^ of 1400 patients confirmed Xofigo’s favourable long-term safety profile. Within its indication, Xofigo has demonstrated consistent efficacy and safety, serving as a reference for newer alpha-emitter therapies like DaRT.

1. TAT is used to target cancer cells more specifically. This requires a guiding molecule that delivers the isotope to the cancer cells,^16^ which can be achieved by conjugating the alpha emitter with mAbs that recognize tumour-associated antigens/receptors (Figure 3B). Such treatment is known as radioimmunotherapy (RIT).^19^ This technique has been clinically approved; one example is [^225^Ac]Ac-PSMA-617, a TAT that targets prostate cancer cells expressing the prostate-specific membrane antigen (PSMA).^20^ 225Ac has a short half-life of 9.92 days. During its decay it releases a 5.83 MeV alpha particle. Following different decay sequences, it emits 4 alpha particles and 2 beta particles, with a total energy release of about 28 MeV, making 225Ac an especially powerful radionuclide.^21^
2. DaRT applies alpha radiation to specific sites, not as a systemically injected solution, as in the 2 other techniques described above. In these seeds, the wires are impregnated with ^224^Ra (Figure 3C).
3. Boron neutron capture therapy (BNCT) (Figure 3D) is not a direct alpha irradiation source; rather, it produces a nuclear reaction that releases high-energy alpha particles with a limited range of 5-9 μm.^22^

### Evidence of the biological advantages of therapy with high LET

LET quantifies the amount of energy transferred to the medium/cells by the incident particle per unit length of its trajectory. Compared with low-LET particles, alpha particles cause high LET, meaning that alpha particles reach a shorter range and have a more intense cytotoxic effect. Studies on animals and cell cultures have shown that, per unit of absorbed dose, the acute biological effects of alpha particles are 3-7 times greater than those caused by external beam or beta radiation.^1^ Therefore, alpha particles have a higher RBE.^23^ The LET of alpha particles ranges from 25 to 230 keV/μm, depending on the particle energy. Their LET is 100-1000 times greater than the average LET of beta particles.^1^ A high LET rapidly dissipates its energy, producing short, dense ionization tracks. Low-LET radiation sources cause less ionization of atoms along their paths, resulting in less densely ionized tracks.^24^ Consequently, alpha particles cause more damage for a given absorbed dose than does low-LET radiation, such as beta particles and gamma rays. Thus, the advantage of alpha irradiation lies in its important local effect, with very little effect outside its very limited range. This could help reduce the dose to healthy sites, such as immune cells. TAT combined with alpha irradiation has been considered to cause more severe and irreversible xerostomia than a beta source does in the treatment of metastatic prostate cancer. However, it seems that this is not true and that both sources cause xerostomia in a dose-dependent manner.^25^ Alpha irradiation is advantageous not only because it has a shorter path length and high LET but also because of the strongly reduced dependency of the damaging effect on oxygenation.^26^

The expanded biological mechanisms of DaRT encompass its therapeutic effects through a combination of direct cytotoxicity and immune modulation, driven by the unique properties of alpha particles: (1) ICD: DaRT induces ICD, characterized by the release of damage-associated molecular patterns (DAMPs) such as HMGB1, ATP, and calreticulin.^27^ (2) Tumour microenvironment modulation: DaRT alters the tumour microenvironment by increasing the infiltration of cytotoxic CD8+ T cells and reducing immunosuppressive populations such as regulatory T cells and myeloid-derived suppressor cells.^28^ (3) DNA damage and repair pathway inhibition: DaRT may overwhelm or suppress key repair pathways, such as homologous recombination mediated by RAD51.^29^ (4) Cytoplasmic and mitochondrial effects: alpha radiation can disrupt mitochondrial membranes, contributing to cell death and potentially generating ROS.^30^ Numerous *in vitro* studies have examined the impact of alpha irradiation. McMahon et al^31^ explored the biological efficacy of various radiation qualities, emphasizing the LET and RBE of heavy charged particles such as alpha radiation compared with conventional X-rays. In a general mechanistic model that utilized linear-quadratic (LQ) models based on the irradiation of hamster cells with varying LETs, the survival curves steepened with increasing LET, indicating that the number of killed cells per Gy was greater for higher LET. The survival curve ultimately became linear at very high-LET levels. Table 1 lists several *in vitro* studies investigating the effects of alpha particle irradiation. The complexity of DNA damage increases with LET. Compared with low-LET irradiation, high-LET irradiation can induce different types of DNA damage, such as clustered DNA or multiple damaged DNA sites. High LET seems to cause more clustered DNA lesions that are more difficult to repair.^32^ Furthermore, the specific activated DNA repair pathways remain unclear.^33^^,^^34^ The cell repair capacity serves as a model to assess the sensitivity of cells to different types of radiation. The inhibition of double-strand DNA break repair has been investigated as an approach to increase the efficacy of radiotherapy.^35^

The following studies compared the efficacy of alpha-particle therapy with that of beta-particle therapy^36^: An ^225^actinium (^225^Ac)-labelled alpha-particle-emitter was more effective in eliminating breast cancer lung metastases in an animal model than Bismuth-213 (^213 ^Bi, primarily an alpha emitter, although it also emits some beta particles during its decay process) and Yttrium-90 (^90^Y), a pure beta-emitter.^37^ The longer physical half-life of ^225^Ac, together with the emission of 4 net alpha particles per decay, resulted in higher absorbed doses. In a second study in cell lines, radionuclide therapy with the alpha-emitter ^225^Ac and the beta-emitter lutetium-177 (^177^Lu) was compared. The degree of DNA DSBs was significantly greater in tumours treated with ^225^Ac (35%) than in those treated with ^177^Lu (21%).^38^

**Figure 2.**
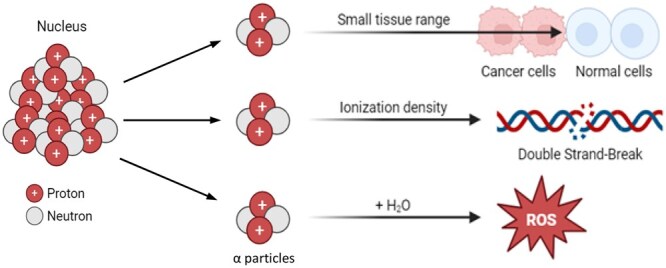
Mechanisms of alpha-emitter radiation therapy. This diagram details the multifaceted actions of high-LET alpha particles on tumour tissue through direct induction of complex DNA double-strand breaks in cell nuclei, extranuclear interactions that generate reactive oxygen species (ROS) and mitochondrial stress, release of damage-associated molecular patterns (DAMPs) that trigger immunogenic cell death and recruitment and activation of dendritic cells and cytotoxic T lymphocytes within the tumour microenvironment.

In a recently published study by Levy et al,^39^ it was shown that alpha irradiation of melanoma cells *in vitro* was more lethal than irradiation with photons and that there was considerable variations in response to alpha irradiation between the different cell lines. And alpha irradiation was more efficient in killing cells than irradiation with photon. The mechanistic pathways of alpha-emitter radiation therapy are schematized in Figure 2, illustrating both direct DNA damage and immune-modulatory effects of high-LET particles.

**Figure 3.**
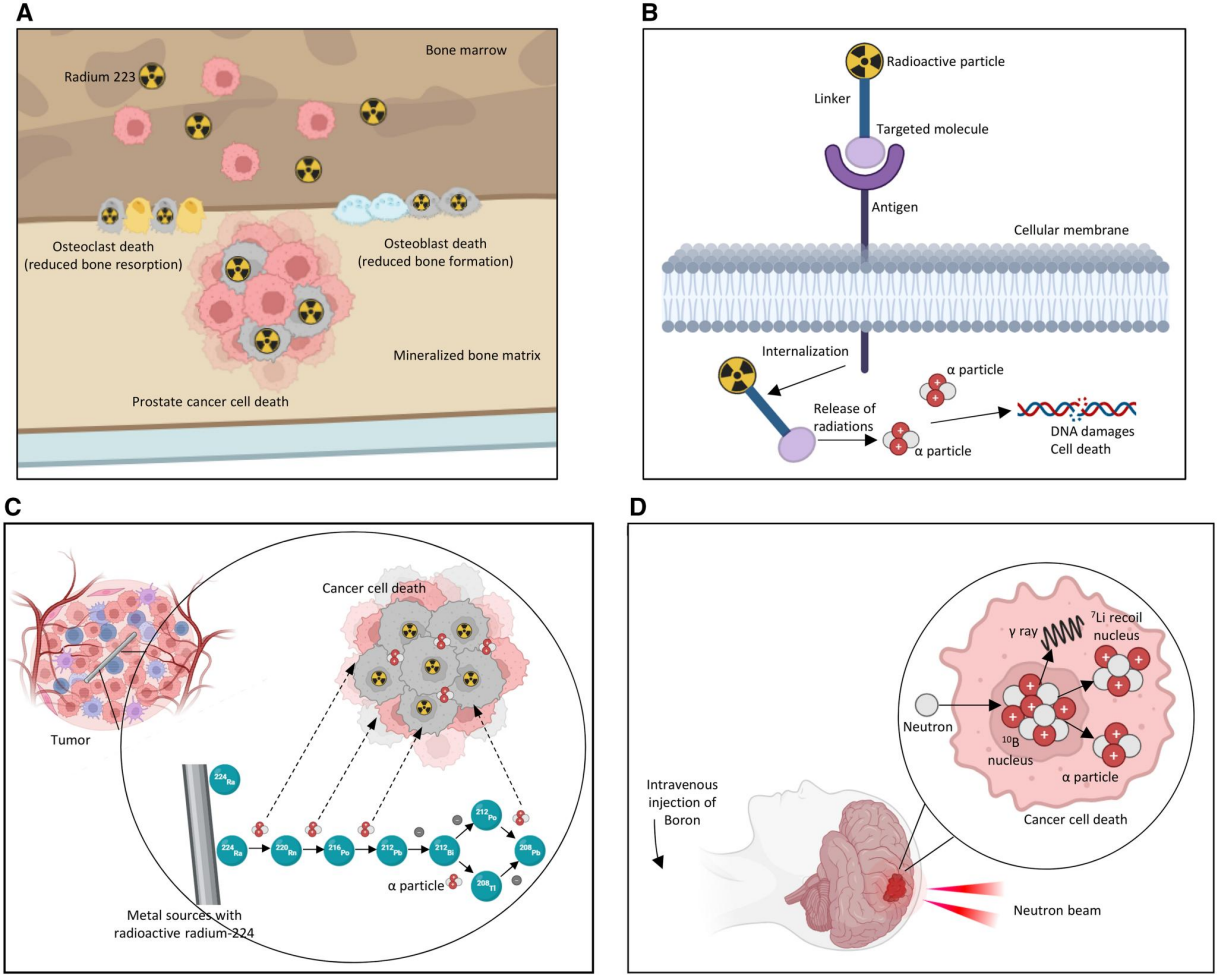
The 4 therapeutic applications of alpha radiation. (A) Radium-223 (Xofigo) in metastatic prostate cancer mimics calcium, localizing to osteoblastic bone metastases. Emitted alpha particles kill tumour cells and induce osteoclast and osteoblast death, reducing both bone resorption and formation in the lesion. (B) Targeted alpha therapy (TAT) with monoclonal antibodies alpha-emitter–conjugated antibodies bind specific tumour-associated antigens on the cancer cell membrane, are internalized, and release α particles that produce lethal DNA damage and subsequent cell death. (C) Diffusing alpha-emitters radiation therapy (DaRT) metal seeds loaded with ^224^Ra are implanted directly into solid tumours. The seeds release ^220^Rn gas, which diffuses into adjacent tumour regions and decays to short-range alpha-emitting daughters, delivering a highly localized, high-LET dose (∼50-100 µm radius). (D) Boron neutron capture therapy (BNCT) patients receive a boron-10 compound that preferentially accumulates in tumour cells. Upon neutron irradiation, ^10^B captures a neutron and splits into ^7^Li and an α particle, causing targeted, high-LET damage within boron-laden cancer cells.

### Immune stimulation

Several clinical and *in vitro* studies have illustrated the potential of combining alpha radiation with other therapies, such as immunotherapy, chemotherapy, or external radiotherapy, to increase the long-term effectiveness of treatment.^40–43^ The immune system is important for sustaining the response to irradiation. Radiotherapy seems to induce in situ vaccination through the release of tumour-associated antigens that provoke an immune system response against cancer and can induce ICD.^44^ To illustrate the effect of radiation, individuals with prostate cancer, an immunologically cold cancer, have low levels of CD8+ T cells and high levels of immunosuppressive cells.^45^ The damage induced by radiation causes the recruitment and maturation of dendritic cells and other effects on the immune system.^45^ One of the problems when comparing preclinical and clinical data is that the available clinical literature is focused mostly on systemic immune responses, whereas preclinical data are limited to local diseases and reactions.^45^ Another problem is that data regarding the effects of radiation on different cell types, such as endothelial cells and fibroblasts, and how they affect the immune response are limited.

Radiation can also induce immunosuppressive effects by inducing dendritic and T cell dysfunction.^45^ In addition to the complexity of the interaction between radiotherapy and the immune system, the effects of different radiation treatments on the immune system depend on the timing of the radiation and immunotherapy as well as on fractionation and total dose.^44^^,^^46^

*In vitro* data have shown that alpha radiation can elicit a stronger antitumor immune response than can low-LET irradiation, resulting in antitumor activity beyond the irradiated site.^47^ A study in a mouse model of cervical cancer showed that dual therapy with methyl jasmonate (MJ) and X-rays or alpha rays requires a lower dose of alpha radiation than X-rays to inhibit cell growth.^48^ MJ exhibited activity against cancer cells both *in vitro* and *in vivo* without affecting normal cells.^49^

If alpha irradiation causes a greater release of these tumour antigens, there may be a stronger reaction of the immune system and greater synergy with the immune system than with conventional or beta irradiation.^44^ However, it is difficult to prove this effect. We found a general lack of comparative studies between different radiotherapy modalities, doses, and their effects on the immune system.^45^ As an exception, in an interesting study from 2023, Guerra Liberal et al^50^ compared irradiation with X-rays, alpha particles, and a combination of both. They concluded that alpha particles may cause fewer but more-complex DSBs, which take longer to repair. Furthermore, at the same dose, alpha irradiation caused more sublethal damage (SLD) than X-rays did. However, pinpointing the clinical significance of this study was difficult. It seems that SLD repair did not differ between the 2 modalities and that a combination of both modalities resulted in a greater RBE. Such an interaction is generally difficult to measure because of the small track of alpha damage. It is generally difficult to analyse the effect of alpha irradiation because of the small number of tracks and the complexity of radiation-induced DNA damage.

### Immune stimulation with DaRT

The scientists involved in DaRT have studied the effects of local alpha irradiation on the immune system. They reported that, in mice, DaRT in combination with an anti-PD-1 (aPD-1) antibody delayed tumour development and induced CD3 and CD8 lymphocyte infiltration into the tumour more efficiently than either monotherapy did.^51^ Moreover, DaRT promoted a “hot” tumour microenvironment and changes in immune suppression that potentiated the aPD-1 blockade-induced effect. Keisari et al^52^ studied the effects of direct alpha particle injection in solid mouse lung cancer. They reported that the immune adjuvant synthetic CpG oligonucleotides (CpG DNA) amplified tumour growth inhibition and reduced pulmonary metastases. CpG DNA can stimulate a potent, orchestrated tumour-specific immune response.^53^

### Does alpha-particle irradiation cause a higher rate of abscopal effects?

Stimulation of the immune system following irradiation can cause shrinkage of untreated cancers that are often distant from the irradiated cancer, although this outcome is considered rare in clinical practice.^47^ This effect is called “abscopal” and was first observed in 1953 by Mole. A very interesting study in xenografted mice revealed that irradiation with high-LET sources, such as Auger electrons and alpha irradiation, has a significant effect outside the directly hit cells (non-targeted effect).^54^ Alpha irradiation might have greater potential for an abscopal effect via stronger activation of the immune system. It has been demonstrated that alpha particles induce ICD, leading to the release of danger-associated molecular patterns (DAMPs), such as Hsp70 and HMGB1. These molecules are known for their role in inflammation and subsequently contribute to the activation of the immune response.^55^ However, elimination of tumour cells by the immune system can lead to the selection of resistant tumour clones.^56^

Clinical evidence of such an abscopal effect from alpha irradiation comes from the report by Bellia et al.^57^ This noteworthy case report has demonstrated that DART may also induce an abscopal effect. In this report, a patient with multiple synchronous squamous cell carcinomas on the skin of the lower limbs had only one lesion treated with DaRT. The primary treated lesion exhibited significant shrinkage, along with a reduction in the size of 2 more distant lesions. This reaction was attributed to an immune-mediated response. One year following the treatment, a complete remission of the treated lesion was observed, as well as spontaneous regression of the untreated distant lesions.

This effect has also been described *in vitro*. Gorin et al^55^ reported activation of the adaptive T cell-dependent immune system after the injection of a vaccine based on alpha-emitting bismuth-213 (^213 ^Bi) particles into a murine colic adenocarcinoma. In this *in vitro* study, the researchers showed that some cells also released DAMPs, which facilitated the activation of dendritic cells. This results in an abscopal effect.

### First clinical results with the DaRT

DaRT is still in early-phase clinical development but has shown enough promise to warrant expansion into multicentre trials. Ongoing studies are exploring DaRT in combination with immunotherapy, chemotherapy, and external beam radiation. Beyond skin and pancreatic cancers, DaRT is being considered for lung, cervical, and gynaecologic tumours, especially where systemic therapies are ineffective or contraindicated.^2^

A recently published pooled analysis of 4 prospective trials including 81 lesions of head and neck or skin cancers in 71 patients with a median follow-up of 14.1 months revealed that the 2-year actuarial local recurrence-free survival rate was 77%. A total of 27% of patients had acute treatment-related toxicity and were treated conservatively. There was no late toxicity after 3 months.^58^ In the initial report of a first-in-human, multicentre clinical study involving 28 patients with squamous cancers of the skin and head and neck, the toxicity observed up to 3 months after DaRT insertion was predominantly manifested as local pain, erythema at the implant site, swelling, and mild skin ulceration. None of the serious side effects were related to the treatment itself.^59^ Furthermore, none of the first 5 patients treated for pancreatic cancer experienced serious adverse events that were judged to be associated with the treatment.^60^ In addition to the ongoing clinical trials targeting skin, head and neck, and pancreatic cancers, DaRT is currently being investigated in various other tumour types using preclinical models. For example, research conducted on orthotopic lung cancer models has demonstrated significant tumour regression following intratumoral DaRT implantation. Similarly, preliminary assessments in glioblastoma and other primary brain tumours—contexts in which brachytherapy has been traditionally employed—have revealed promising antitumor effects with minimal off-target toxicity. These findings suggest that DaRT’s highly localized, high-LET alpha particle delivery may extend therapeutic benefits to cancers beyond those currently under clinical investigation.

DaRT has been investigated in cervical and gynaecologic cancers as well as in prostate cancer due its accessibility and large experience with brachytherapy in these cancers in general.

## Conclusion

It is well established that alpha irradiation has a higher RBE and is better able to spare healthy tissue because of the smaller range of irradiation and the lower dependence on oxygenation. DNA damage seems to be more complex and, therefore, more difficult to repair and could induce a stronger immune reaction.

So far, preclinical and clinical findings indicate a paradigm shift in local cancer therapy with the introduction of DaRT. Its distinct advantages, including potent cytotoxicity, spatial precision, and synergy with immunotherapies, position DaRT as a promising innovation. While long-term clinical validation is still underway, DaRT’s application across various cancers continues to advance. By combining focused alpha delivery, strong local tumour control, and immune activation, this innovative approach holds promise for improving outcomes in solid tumours that currently receive limited benefit from systemic therapies. Its feasibility in skin cancers and promising results in pancreatic cancers, as well as the observed abscopal effect, extend the clinical results beyond theoretical promise. DaRT could be utilized in pre-treated inoperable or radio-recurrent cancers where the exposure of previously irradiated healthy tissue is critical. Future research and clinical applications may focus on integrating DaRT with immunotherapy such as checkpoint blockade refining dosimetry strategies, or evaluating its efficacy in immune-evasive tumours.

On the other hand, the range of high LET is smaller and therefore results in a smaller number of DSBs. Because of the small number of tracks that it generates and the difficulty in evaluating the clinical impact of more-complex DNA damage, alpha irradiation seems promising but has yet to prove its clinical advantage. Certain aspects of alpha irradiation remain incompletely understood. The full biological complexity, especially regarding immune modulation, repair pathway engagement, and systemic effects, requires further elucidation. While the radiobiological efficacy of high-LET alpha irradiation is well supported by preclinical data and early clinical experience, its broad clinical validation remains in progress. Other significant topics, such as how intratumoral factors might influence the efficacy of DaRT remain inadequately understood.
